# Effect of oral lactulose on clinical and immunohistochemical parameters in patients with inflammatory bowel disease: a pilot study

**DOI:** 10.1186/1471-230X-7-36

**Published:** 2007-09-04

**Authors:** Anne Hafer, Sigrid Krämer, Swantje Duncker, Martin Krüger, Michael P Manns, Stephan C Bischoff

**Affiliations:** 1 Department of Gastroenterology, Hepatology and Endocrinology, Medical School of Hannover, Carl-Neuberg-Str. 1, 30625 Hannover, Germany; 2 Department of Nutritional Medicine, University of Hohenheim, Fruwirthstr. 12, 70593 Stuttgart, Germany; 3 Brain-Body Institute, St. Joseph's Healthcare, McMaster University, 50 Charlton Avenue E., Hamilton, ON, L8N 4A6, Canada

## Abstract

**Background:**

The prebiotic potential of lactulose is well established and preclinical studies demonstrated a protective effect of lactulose in murine models of colitis. The aim of the present study was to investigate the clinical and histological efficacy of lactulose in patients with inflammatory bowel disease (IBD), for which probiotic therapy yielded promising results.

**Methods:**

Patients were treated with standard medication alone or combined with 10 g lactulose daily as adjuvant therapy for 4 months. Clinical efficacy of treatment was assessed using clinical activity indices, a quality of life index (IBDQ), endoscopic scores, defecation frequency and monitoring corticosteroid medication. Orsomucoid, alpha1-antitrypsin and other laboratory parameters were determined. In addition, in some participants colonic biopsies were analyzed with haematoxylin-eosin staining or with antibodies against HLA-DR, CD68, IgA and CD3, and evaluated systematically. All measurements were performed both at enrolment and at the end of the trial.

**Results:**

14 patients presenting ulcerative colitis (UC) and 17 patients presenting Crohn's disease (CD), most of them in a clinically active state, were enrolled in this pilot study. After 4 month no significant improvement of clinical activity index, endoscopic score or immunohistochemical parameters was observed in CD or UC patients receiving lactulose in comparison to the control group. However, significant improvement of quality of life was observed in UC patients receiving lactulose compared to the control group (p = 0.04).

**Conclusion:**

The findings of the present pilot study indicate that oral lactulose has no beneficial effects in IBD patients in particular with regard to clinical activity, endoscopic score or immunohistochemical parameters. The importance of the beneficial effect of lactulose in UC patients regarding the quality of life needs further evaluation in larger controlled clinical trials.

**Trial registration:**

Current Controlled Trials ISRCTN92101486

## Background

Inflammatory bowel diseases (IBD), commonly referred to as Crohn's disease (CD) and ulcerative colitis (UC) are recurrent aggressive inflammatory conditions of multifactorial etiology, which to date are not well understood. Interactions of genetic background, disturbance of the mucosal barrier, dysregulation of intestinal immune responses as well as bacterial and other environmental factors were found to play a role in the development of IBD.

In this context, the mucosal barrier is a key factor, since its disturbance usually precedes the onset of IBD [1]. At the same time, a change of intestinal flora can be detected, specifically with regard to adherent bacteria [2,3]. In animal models of colitis, it has been shown that IBD does not develop in a germ-free environment [4]. In human ulcerative colitis, exacerbations of intestinal inflammation can be prevented by probiotic bacteria, possibly because they inhibit pathogenic bacteria by either growth or adherence inhibition and competition, respectively [5].

Previous medical treatment of IBD has predominantly focused on nonspecific suppression of the inflammatory process. Antibiotics can selectively decrease tissue invasion of bacteria and eliminate aggressive bacterial species [6]; however, such treatment is not sustaining and often accompanied by substantial side effects. The alteration of the intestinal flora by probiotics (beneficial bacterial species) and prebiotics (poorly absorbed dietary oligosaccharides) may offer an alternative therapeutic approach. Such substances are capable of modulating the intestinal flora in IBD, resulting in a predominance of beneficial *Lactobacillus *and *Bifidobacterium *species and in clinical benefit [7,8].

Some controlled clinical trials have been conducted regarding probiotic therapy in UC, showing that the probiotic bacterial mixture VSL#3 is effective in preventing pouchitis [9-11], and that the probiotic *E. coli Nissle *1917 prevents relapses of active UC [12-14]. In CD, probiotic trials yielded inconsistent results [15-20]. A few pilot studies suggested that *E. coli Nissle *1917 is beneficial in tapering steroids in CD [15], that *Lactobacillus *GG may be effective in children with CD [16,17], and that Saccharomyces boulardii may be beneficial in adult CD [18,19].

Prebiotics stimulate the growth and metabolism of protective commensal enteric bacteria, as well as the production of short chain fatty acids. The net result of prebiotic administration is theoretically similar to administering probiotic bacteria, however, the effect of prebiotics on the patient's flora can continue for several weeks after cessation of treatment [21], while the effect of probiotics is generally shorter. To date, only a few clinical trials on prebiotics in IBD have been conducted. In one open-label trial, prebiotic germinated barley foodstuff was shown to reduce clinical and endoscopic activity in patients with UC [22]. The prebiotic potential of lactulose is well established [23]. In murine models of colitis, lactulose decreases the number of adherent and translocated bacteria as well as histologically detectable inflammation [24]. Furthermore, lactulose ameliorated DSS-induced colitis in rats in a dose-dependent manner [25].

In summary, trials in both experimental colitis and human IBD suggest a potential role of probiotic bacteria and prebiotic products for treatment of IBD. The aim of the present study was to investigate clinical effects of lactulose in IBD patients in a pilot study to further assess if there is a rationale for large scale clinical trials. In addition, immunohistochemical tests were performed to study the potential effect of lactulose on mucosal cell infiltration.

## Methods

### Patients and study design

52 patients of the Department of Gastroenterology at the Hannover Medical School were recruited and randomised between August 2000 and July 2003. The study was approved by the Ethical Committee of the Hannover Medical School. All procedures were in accordance with the Declaration of Helsinki. To be included in the trial, patients had to present IBD. In most of them the clinical activity was confirmed by elevated Clinical Activity Index (CAI) scores in UC or elevated Crohn's Disease Activity Index (CDAI) scores. The diagnosis of IBD was confirmed by classical clinical and endoscopic means according to the German and Austrian guidelines for UC and CD [26-28]. Clinical parameters were obtained from all patients. In addition to the clinical parameters in some patients immunohistochemical parameters were analyzed. Therefore biopsies were collected and analyzed at enrolment and at the end of the study.

The trial was conducted as a prospective, randomised and controlled pilot study with a parallel-group design. Patients in the control group received standard medication according to the recommendations of the guidelines [29-31]. Patients in the lactulose group received standard medication plus 15 ml lactulose syrup (containing 10 g lactulose) daily as adjuvant therapy. All investigations were performed at enrolment and at the end of the 4-months trial period.

### Evaluation of clinical efficacy

#### Clinical activity indices

Well-established activity indices were used to assess the activity of the inflammatory process. For UC, we employed the CAI according to Rachmilewitz [32]. High point CAI-scores correspond with a high activity of inflammation, with remission being defined as a CAI score of ≤ 4. Since erythrocyte sedimentation rate (ESR) is not routinely measured at our hospital, C-reactive protein (CRP) values reflecting similar activity were used as a proxy [33]. For CD the CDAI according to Best [34] and the Severity and Activity Index (SAI) was used [35]. High point CDAI scores indicate a high activity of inflammation, a decrease of more than 100 points implies clinical improvement, and remission is defined as a CDAI score of ≤ 150 [36]. In addition, Defecation frequency was documented by all patients over a period of one week using an appropriate diary for evaluation of clinical activity of the disease.

#### Quality of life index

To assess quality of life, the German version of the Inflammatory Bowel Disease Questionnaire (IBDQ) was used [37]. It includes 32 questions concerning mood, pain, and limitations in daily life. Scores between 32 and 224 points may be reached. High values indicate a better quality of life, and remission is defined as an IBDQ score of > 190. All questionnaires were completed by the patients retrospectively at presentation.

#### Medication

To assess clinical efficacy, required corticosteroid medication was documented semiquantitatively (no corticosteroids/≤ 5 mg prednisolone equivalent/> 5 mg prednisolone equivalent).

#### Endoscopic scores

Local inflammation was assessed by endoscopic scores using an ordinal scale, high values indicating high activity of inflammation. For UC the Baron Scale, discriminating normal mucosa/erythem/erosions/ulcerations and spontaneous bleeding was applied, with scores ranging from 0 to 3 points [38]. For CD a score similar to the Rutgeerts Score was used, assessing criteria such as normal mucosa/< 5 aphthae/> 5 aphthae or skip lesions/diffuse aphthae/diffuse inflammation with ulcerations, nodules, and strictures, with scores ranging from 0 to 4 points [39].

#### Laboratory parameters

In addition to clinical findings, a standard set of laboratory parameters was used to assess disease state and/or activity.

In blood, haemoglobin (Hb) and haematocrit values were determined to calculate activity indices. From serum, we determined orsomucoid (Ors), albumin (Alb), and the immunoglobulins IgG, IgA and IgM. The "Factor Score" was calculated according to the formula 1/2 × Hb (g/dl) + Alb (g/l) - 4 × Ors (g/l) - 37. Positive values indicate remission, negative values indicate an acute episode of inflammation [40].

In feces samples, α_1_-antitrypsin and the pH were measured. The α_1_-antitrypsin concentration is a marker for intestinal loss of protein and impaired intestinal permeability. In CD patients, this marker has been established as an activity marker [41]. The pH measurements were used to verify patient compliance, since lactulose administrations causes a decrease in fecal pH values [21].

### Histological and immunohistochemical evaluation

During colonoscopy or surgical intervention, mucosal biopsies of a size of 1 × 1 × 2 mm^3 ^were taken from areas showing maximal inflammation. Haematoxylin-eosin (HE) stainings and immunohistochemical (IHC) stainings were performed.

For each biopsy the percentages of lamina propria eosinophils and neutrophils as well as of intraepithelial lymphocytes were assessed. To evaluate the inflammatory activity, a HE Score including epithelial integrity, mucosal architecture, mononuclear cell infiltration, neutophil-granulocytic cell infiltration and crypt abscesses was calculated analogous to the scoring system for IBD described by Geboes et al. [42]. High points correspond with high inflammatory activity.

Immunohistochemical stainings were performed using the labeled-streptavidin-biotin (LSAB) method for determination of mucosal counts of T-cells, IgA-producing cells and macrophages as well as the extent of cell activation. Primary antibodies included (1) anti-CD3 (monoclonal mouse IgG2a, Biocarta, Hamburg, Germany) to mark mucosal T lymphocytes, (2) anti-IgA antibody (α-Heavy Chain) Ab-1 (monoclonal mouse IgG1κ, Biocarta, Hamburg, Germany) to identify IgA producing plasma cells, (3) anti-CD68 (monoclonal mouse IgG1κ, Zymed Lab., San Francisco, USA) to detect macrophages and (4) antihuman HLA-DR (monoclonal mouse IgG1κ, DAKO, Glostrup, Denmark), a general marker of cell activation [43-46]. Human-absorbed, biotinylated, affinity-purified secondary antibodies were introduced according to manufacturer instructions (Histostain-Plus Kit, Zymed, San Franzisco, USA). Slides were counterstained with Mayer's haemalaun (Merck, Darmstadt, Germany). For each staining, an appropriate isotype control antibody was included. To calculate the percentage of CD68, IgA and CD3, one thousand lamina propria cells per biopsy were counted. The evaluation of HLA-DR expression was performed semiquantitatively since it was not possible to differentiate between all individual cells. The semiquantitative analysis was performed using a score adapted from Beaugerie et al [47]. All counts were performed in a blinded manner by the same investigator.

### Statistics

Data are presented as means ± standard error of the mean (SEM), unless indicated otherwise. Mann-Whitney-U test were used to evaluate the comparability of the study groups at baseline. To compare the data of the lactulose group with those from the control group, the difference of the mean values (value at the end of the study minus value at the start of the study) for each group was calculated. For comparison, the unpaired T-Test was employed. The T-test for paired samples was used to uncover potential differences between mean values at start and end of the study. The statistical analysis of ordinal data (histological and immunohistological data) was performed using the Mann-Whitney-U test for unpaired samples and the Wilcoxon matched pairs test for paired groups. A p ≤ 0.05 was considered significant, p values > 0.05 to 0.10 were interpreted as tendencies. All statistical analysis was performed using the software package SPSS 12.0.

## Results

### Study population

**Figure 1:**
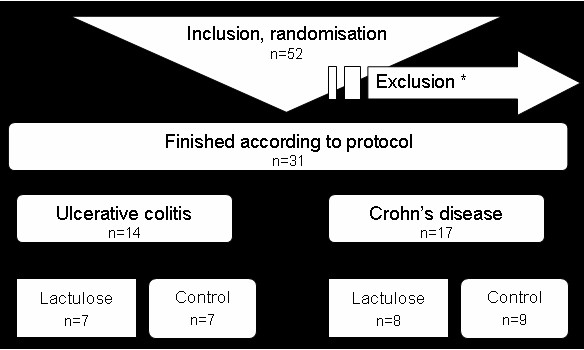
**Study design**. *Criteria for exclusion of patients were: out of reach (10), surgery (4), protocol too demanding (3), drug intake not reliable (3), different study during study period (1).

A total of 31 IBD patients completed the study according to protocol, 14 of whom presented UC, and 17 CD. 7 UC and 8 CD patients had been randomly assigned to the lactulose groups (L), with the remaining 7 UC and 9 CD patients belonging to the control groups (C) (Figure 1). The majority of patients enrolled in this study were hospitalized because of symptoms of active disease. 5 patients enrolled in this study (4 UC patients of the control group and 1 UC patient of the lactulose group) were ambulant patients who came because of symptoms and underwent a colonoscopy. Demographic and clinical characteristics of the patients at baseline are shown in table 1. The participants received standard medication during the whole study. Medication at baseline is also shown in table 1. Duration of participation among the different groups show no statistical differences. In addition to the clinical parameters in 20 patients, 9 of whom presented UC the effect of oral lactulose on immunohistochemical parameters was analyzed. Therefore biopsies were collected and analyzed at enrolment and at the end of the study.

**Table 1:** Baseline characteristics of the study groups

	Ulcerative colitis		Crohn's disease	
	
	Lactulose (n= 7)	Control (n= 7)	Lactulose (n= 9)	Control (n= 8)
Age*	38.8 ± 12.4	38.1 ± 8.8	30.7 ± 7.0	33.5 ± 11.7
Female sex	3	4	5	4
Disease duration (years)*	8.3 ± 12.2	9.3 ± 5.0	5.0 ± 5.4	6.7 ± 9.8
Disease Activity (CAI/CDAI)*	10.3 ± 5.9	10.4 ± 11.2	195.5 ± 148.4	273.9 ± 112.9
IBDQ*	123 ± 47.0	132 ± 58.4	136 ± 42.7	134 ± 43.3
Defecation/week*	37 ± 24.3	36 ± 24.1	39 ± 30.4	45 ± 23.3
Duration of participation*	3.7 ± 1.5	4.8 ± 2.5	5.0 ± 3.1	4.3 ± 2.2
				
Medication				
Prednisolone equivalents > 5 mg	5	2	6	6
Prednisolone equivalents ≤ 5 mg	0	0	1	3
5-ASA	7	4	6	3
Immunosuppressive drugs	1	1	3	2
Antibiotics	0	3	5	4

*(Mean ± SD)

### Clinical data

#### Clinical activity indices

**Figure 2:**
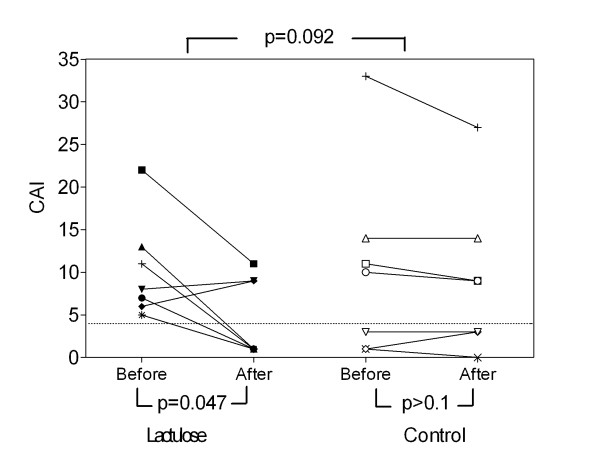
**Clinical activity in ulcerative colitis**. The clinical activity index (CAI) was assessed. Values below 4 (dotted line) indicate remission. Baseline and final scores after 4 months of supplementary lactulose therapy versus standard medication alone are shown.

**Figure 3:**
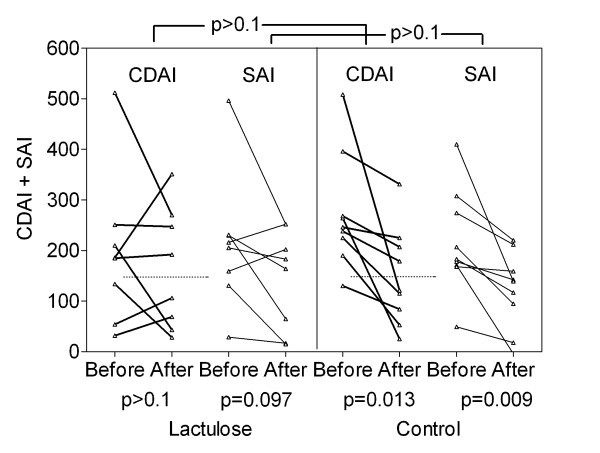
**Clinical activity in Crohn's disease**. Clinical activity was measured using the CDAI and SAI scores (see Methods). Remission is indicated by a CDAI ≤ 150 (dotted line). Baseline and final scores after 4 months adjuvant lactulose therapy (lactulose) versus standard medication alone (control) is shown.

No significant improvement of clinical activity index was observed in CD (p > 0.1) or UC (p = 0.092) patients receiving lactulose in comparison to the control group (Figure 2). In UC, clinical activity of the disease could be reduced within the lactulose group. In this group, the CAI improved by 5.6 ± 2.3 from 10.3 ± 2.2 to 4.7 ± 1.8 (p = 0.047), and 4 out of 7 patients reached remission. In the control group, the CAI did not change (start of therapy 10.4 ± 4.2, end of study 9.3 ± 3.6), none of 4 patients with active disease reached remission (Figure 2). In CD, both the CDAI and SAI showed improvement only in the control group. Without lactulose, CDAI improved by 125 ± 39 from 274 ± 38 to 149 ± 32 (p = 0.013), SAI improved by 95 ± 28 from 216 ± 34 to 122 ± 26 (p = 0.009). In the lactulose group, the CDAI did not change significantly (start of therapy 196 ± 53, end of study 163 ± 42) (Figure 3).

#### Quality of life index

**Figure 4:**
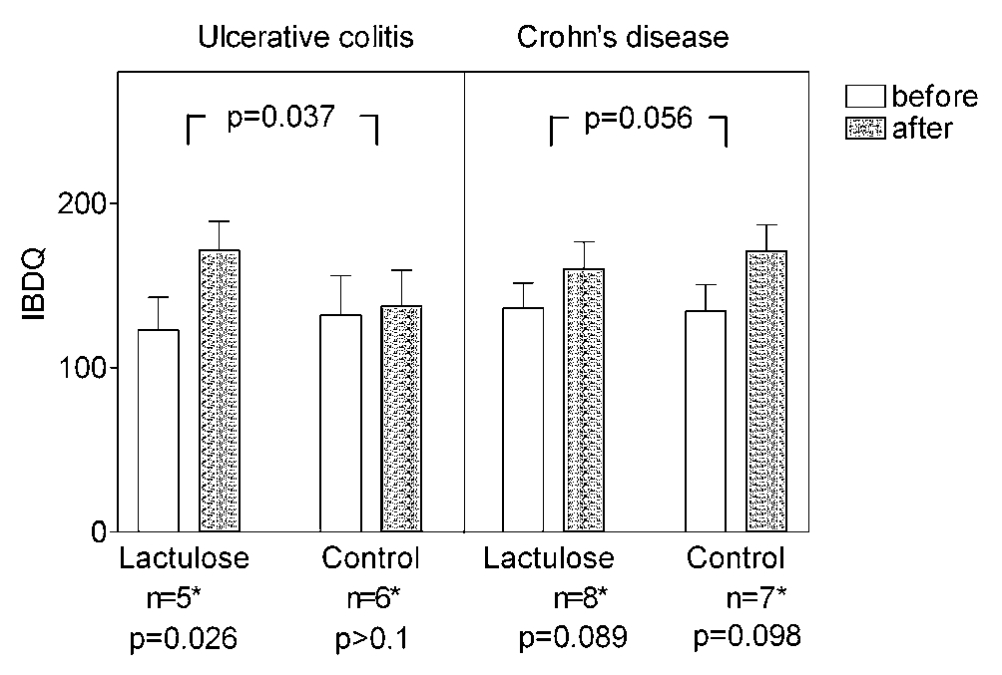
**Quality of Life**. Assessment of a standardized and validated IBD questionnaire, evaluated by IBDQ (IBDQ, see Methods), consisting of 32 questions for mood, pain, and limitations in daily life. High values indicate a better quality of life; remission is defined as an IBDQ score of > 190. Means ± SEM for baseline and final scores after 4 months of supplementary lactulose therapy versus standard medication alone are shown. *Not all patients responded to the questionnaire.

In the UC lactulose group, the IBDQ score increased by 48 ± 14 from 123 ± 20 to 171 ± 18 (p = 0.026), in contrast, the IBDQ did not change significantly in the UC controls (start of therapy 132 ± 24, end of study 138 ± 22). The improvement in the lactulose group proved to be statistically significant compared to controls (p = 0.037). In CD, IBDQ increased by 24 ± 12 from 136 ± 15 to 160 ± 17 (p= 0.089) in the lactulose group, and by 37 ± 19 from 134 ± 17 to 171 ± 16 (p = 0.098) in the control group (Figure 4).

#### Medication

During the study period, corticosteroid medication could be reduced in many patients. While 4 of 5 UC patients receiving corticosteroids in the lactulose group could reduce their corticosteroid medication (p = 0.063), 1 of 2 UC patients in the control group could reduce it. In CD, 1 of 7 patients receiving corticosteroids in the lactulose group could reduce the dosage of corticosteroids, while one had to increase it, compared to 3 of 9 patients who could reduce their medication in the control group. However, there were no significant differences between the groups.

#### Endoscopic scores

The endoscopic scores showed no change between the treatment groups (CD control group from 2.3 ± 0.9 to 2.4 ± 0.9, lactulose group 2.6 ± 0.7 to 1.8 ± 1.1; UC control group from 1,7 ± 0,6 to 2,4 ± 0,4, lactulose group from 1,4 ± 0,5 to 2.0 ± 0.0)

#### Defecation frequency

**Figure 5:**
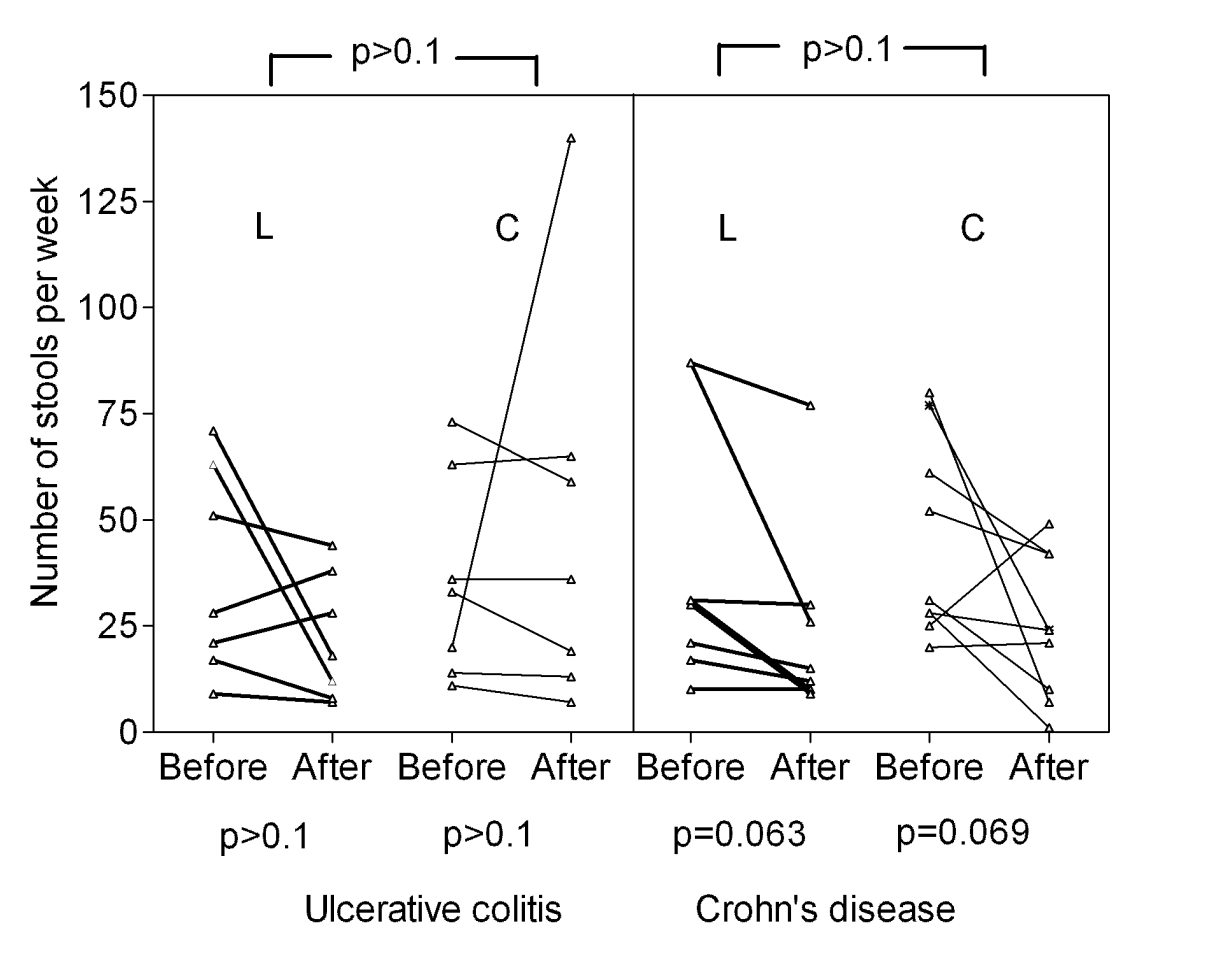
**Bowel movements**. Frequency of defecation in UC and CD per week in the lactulose group (L) and control group (C). Baseline and final numbers after 4 months supplementary lactulose therapy versus standard medication alone are shown.

No significant change in defecation rate was found for the UC groups. In CD, defecation rate showed a trend to decrease both in the lactulose group by 16 ± 7 from 39 ± 11 to 24 ± 8 (p= 0.063), and in the control group by 20 ± 10 from 45 ± 8 to 24 ± 6 (p= 0.069). (Figure 5).

#### Safety

Four out of 15 patients receiving lactulose had minor side effects. In UC, one patient reported an increased defecation frequency and slight flatulence, another suffered from abdominal pain because of meteorism. In CD, one patient reported on slight flatulence; another one on decreased stool consistency. Despite these facts all patients completed the study according to protocol.

### Laboratory parameters

#### Blood

Immunoglobulin (Ig) concentrations in UC and CD patients were not changed by lactulose (table 2). The Factor score defined in the 'Method' section showed improvements in both CD groups (Lactulose group: by 8.4 ± 3.0 from -7.0 ± 3.1 to 1.3 ± 2.6 (p = 0.044); Control group: by 6.0 ± 2.5 from -4.7 ± 3.1 to 1.3 ± 2.0 (p = 0.028); however, there was no statistically significant difference between the treatment groups (table 2).

**Table 2:** Developing of blood and fecal parameters

	Ulcerative colitis		Crohn's disease	
	
	Lactulose (n= 7)	Control (n= 7)	Lactulose (n= 9)	Control (n= 8)
Immunoglobulin G (g/l)*				
Before	12.3 ± 1.3	14.0 ± 2.0	12.9 ± 4.6	11.8 ± 3.4
After	12.9 ± 1.2	13.3 ± 2.0	11.8 ± 4.5	11.6 ± 4.1
Immunoglobulin A (g/l)*				
Before	3.0 ± 0.5	2.8 ± 0.4	4.6 ± 3.4	3.6 ± 1.1
After	3.0 ± 0.7	2.3 ± 0.3	4.3 ± 3.4	3.6 ± 1.3
Immunoglobulin M (g/l)*				
Before	1.9 ± 0.2	2.4 ± 0.8	1.9 ± 1.7	1.7 ± 0.5
After	2.1 ± 0.4	2.3 ± 0.7	1.9 ± 1.7	1.8 ± 1.0
Blood activity factor^*,†^				
Before	-2.9 ± 1.9	2.9 ± 2.4	-7.0 ± 3.1	-4.7 ± 3.1
After	5.4 ± 2.8	5.7 ± 1.2	1.3 ± 2.6^‡^	1.3 ± 2.0^‡^
Fecal α1-antitrypsine*				
Before	69.5 ± 17.6	56.8 ± 14.4	79.8 ± 9.9	66.5 ± 9.7
After	27.8 ± 9.0	50.0 ± 12,7	55.3 ± 14.1	35.2 ± 11.4^‡^
Fecal pH*				
Before	6.4 ± 0.2	6.8 ± 0.1	6.8 ± 0.2	6.7 ± 0.7
After	7.0 ± 0.3	6.8 ± 0.1	6.6 ± 0.4	6.6 ± 0.7

* (Mean ± SEM)

^† ^Factor score: 1/2 × Hb (g/dl) + Alb (g/l) - 4 × Ors (g/l) - 37.

^‡ ^p < 0.05 in comparison to the baseline

#### Feces

In UC treated with lactulose, α_1_-antitrypsin concentration decreased (UC/L: -41.8 ± 15.6 from 69.5 ± 17.6 mg/dl to 27.8 ± 9.0 mg/dl; p = 0.076). In UC control, no change was observed (UC/C: +7.9 ± 15.4 mg/dl; n. s.). In CD, the control group showed a decrease by -31.4 ± 9.5 mg/dl from 66.5 ± 9.7 mg/dl to 35.2 ± 11.4 mg/dl (p = 0.016). In the lactulose group we found a similar tendency, albeit not statistically significant (table 2). Differences between the treatment groups did not prove to be statistically significant. pH values tended to increase in the UC lactulose group, but not in the respective control group (table 2).

### Histological and immunohistological data

#### Haematoxylin-eosin (HE) staining

**Figure 6:**
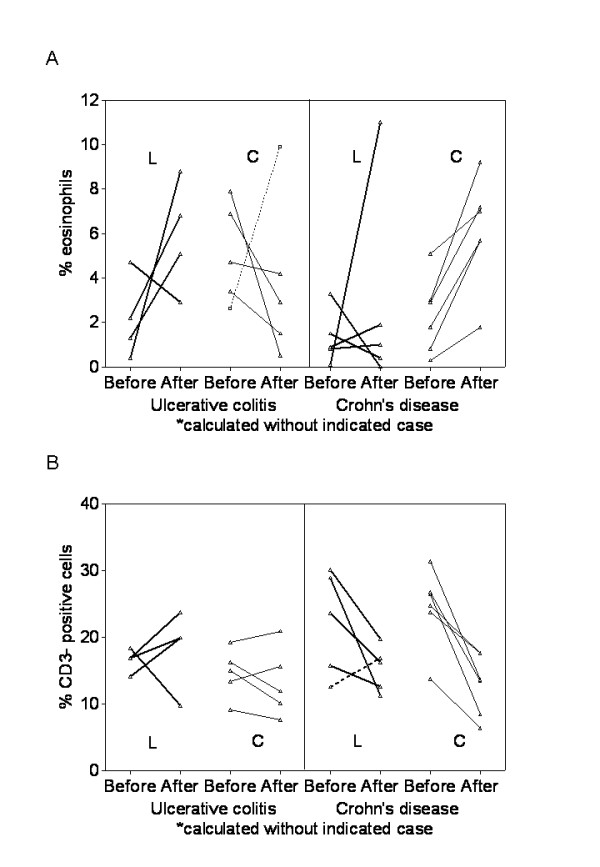
**Immunohistochemistry**. Eosinphil granulocytes (panel A) and CD3 positive lymphocytes (panel B), expressed as percentage of lamina propria cells, were quantified in UC and CD. Baseline and final values after 4 months of supplementary lactulose treatment (L) versus standard medication (controls, C) alone are shown.

The percentage of eosinophils in relation to intestinal lamina propria cells showed a tendency to decrease in the control group of UC (p = 0.066). In CD, the control group showed a significant increase (p = 0.028), but no consistent changes were observed in the lactulose group (Figure 6A). The percentage of neutrophils, intraepithelial lymphocytes and the HE score did not show consistent changes during the study period and no statistically significant differences could be found between the groups.

#### Immunohistological staining

The number of CD3 positive cells decreased in CD patients in the course of the study, reaching significance in the control group (p = 0.028), but not in the lactulose group (p= 0.068). No consistent changes were observed in UC (Figure 6B). Analysis of CD68+ and Ig A+ cells as well as HLA-DR expression revealed no significant changes related to the supplementary lactulose treatment.

## Discussion

The present pilot study was conducted to investigate clinical and immunohistological effects of lactulose in IBD patients. Maybe due to the low number of patients enrolled in this study no beneficial effects in IBD patients with regard to clinical activity, endoscopic score, serum markers, or immunohistochemical parameters could be observed. However, quality of life index (IBDQ) was improved by lactulose at a dosage of 10 g per day as adjuvant therapy to standard medication in UC patients. Since this pilot study is not a placebo controlled study, we can not rule out a placebo effect in the lactulose group. The beneficial effect in clinical activity in UC patients within the lactulose group is subject to review in larger trials.

Previous clinical studies report substantial benefit of particular pre- and probiotics in UC [9-14], but not in CD [15-20]. Fellermann et al. [48] described a defensin deficiency in IBD, which could lead to an impaired intestinal barrier function and subsequently to the initiation of IBD. In UC, defensins are reversibly down-regulated for unknown reasons, whereas in CD, defensin expression is permanently impaired because of genetic alterations. Therefore, probiotics such as *E.coli Nissle *1917 can reverse the decrease in defensin expression in UC, but not in CD. Possibly, lactulose, similarly to the probiotic, could also upregulate defensin expression provided that no genetic mutations have occurred. These mechanisms fit with the observations of Szilagyi et al. [49] who reported that lactulose had a less pronounced effect on the intestinal flora in IBD patients than in healthy controls. In particular, UC patients showed a trend towards adaptation of the colonic flora, while no adaptation was observed in CD. This is likely caused by an increased intestinal permeability, resulting in lactulose absorption, which could further explain the ineffectiveness of lactulose in CD.

The clinical benefit we observed in UC within the lactulose group was not confirmed on the endoscopic level, possibly because the correlation between endoscopic scores and clinical activity is known to be weak. In CD, only one third of patients with corticosteroid-induced clinical remission gained normalisation of the endoscopic changes [50], whereas in UC, this correlation is known to be closer [51]. Moreover, one should consider the fact that an endoscopically normal intestine can still present microscopic inflammatory alterations [52].

It is important to note that the defecation frequency was not different between the lactulose groups and controls. Considering the laxative effect of lactulose in constipation [53], an increase of defecation rates could have been expected in the lactulose groups. Instead, we observed a trend towards decreased bowel movements in all CD patients, independent of the lactulose medication. In UC patients, the number of bowel movements did not change, neither in the lactulose group nor in the control group. We therefore conclude that at dosages administered here (10 g/d), lactulose does not increase defecation rates in IBD patients. Four out of 15 patients experienced some mild to moderate side effects possibly related to lactulose (2× flatulence, 1× increase in the number of bowel movements, 1× both). Overall, the administered dosage of lactulose was well tolerated, with no serious adverse effects being observed.

Laboratory parameters (biochemical and immunohistological markers) yielded only few conclusive results. Lactulose was reported to modify systemic immune responses in a murine model; however, such findings were not confirmed by our immunoglobulin measurements. Interestingly, eosinophil counts in the mucosa were elevated in UC patients receiving lactulose, but not in the corresponding control group. In recent studies, eosinophilia has been associated with healing phases following chronic inflammation. In CD, we made an opposite observation, once more confirming the clinical data that suggested benefit of lactulose only in UC. Other histological and immunohistochemical data were inconclusive or could not be clearly related to the additional treatment with lactulose.

A critically issue of the study is the variation between the groups in prestudy clinical characteristics. For example, the high dosis of prednisolon in 5 UC patients receiving lactulose compared to 2 patients of the control group. The dosis of prednisolon has been changed directly before enrollment in the study. On this account, we can not exclude that the medication is an additional reason for the observed improvement within the lactulose group in UC patients.

## Conclusion

In conclusion, our present pilot study indicates that oral lactulose has no beneficial effects in IBD patients. For sure, a randomized placebo-controlled trial is currently underway to confirm these observations.

## Competing interests

The author(s) declare that they have no competing interests.

## Authors' contributions

Anne Hafer recruited the patients, collected the data and performed the histological and immunohistochemical studies. Furthermore, she analysed the data and wrote the manuscript together with S. Krämer and S. C. Bischoff. Swantje Duncker gave professional support regarding methods of immunohistochemistry. Martin Krüger collected biopsy specimen. Michael P. Manns provided equipment and laboratory space, and he participated in interpretation of results, Stephan C. Bischoff acted as PI of the study, he supervised A. Hafer et al. during the study. All authors read and approved the final manuscript.

## Pre-publication history

The pre-publication history for this paper can be accessed here:



## References

[B1] Irvine EJ, Marshall JK (2000). Increased intestinal permeability precedes the onset of Crohn's disease in a subject with familial risk. Gastroenterology.

[B2] Swidsinski A, Ladhoff A, Pernthaler A, Swidsinski S, Loening-Baucke V, Ortner M, Weber J, Hoffmann U, Schreiber S, Dietel M, Lochs H (2002). Mucosal flora in inflammatory bowel disease. Gastroenterology.

[B3] Kleessen B, Kroesen AJ, Buhr HJ, Blaut M (2002). Mucosal and invading bacteria in patients with inflammatory bowel disease compared with controls. Scand J Gastroenterol.

[B4] Madsen KL, Malfair D, Gray D, Doyle JS, Jewell LD, Fedorak RN (1999). Interleukin-10 gene-deficient mice develop a primary intestinal permeability defect in response to enteric microflora. Inflamm Bowel Dis.

[B5] Setoyama H, Imaoka A, Ishikawa H, Umesaki Y (2003). Prevention of gut inflammation by Bifidobacterium in dextran sulfate-treated gnotobiotic mice associated with Bacteroides strains isolated from ulcerative colitis patients. Microbes Infect.

[B6] Cummings JH, Macfarlane GT, Macfarlane S (2003). Intestinal bacteria and ulcerative colitis. Curr Issues Intest Microbiol.

[B7] Sartor RB (2004). Therapeutic manipulation of the enteric microflora in inflammatory bowel diseases: antibiotics, probiotics, and prebiotics. Gastroenterology.

[B8] Shanahan F (2005). Physiological basis for novel drug therapies used to treat the inflammatory bowel diseases I. Pathophysiological basis and prospects for probiotic therapy in inflammatory bowel disease. Am J Physiol Gastrointest Liver Physiol.

[B9] Gionchetti P, Rizzello F, Helwig U, Venturi A, Lammers KM, Brigidi P, Vitali B, Poggioli G, Miglioli M, Campieri M (2003). Prophylaxis of pouchitis onset with probiotic therapy: a double-blind, placebo-controlled trial. Gastroenterology.

[B10] Gionchetti P, Rizzello F, Venturi A, Brigidi P, Matteuzzi D, Bazzocchi G, Poggioli G, Miglioli M, Campieri M (2000). Oral bacteriotherapy as maintenance treatment in patients with chronic pouchitis: a double-blind, placebo-controlled trial. Gastroenterology.

[B11] Mimura T, Rizzello F, Helwig U, Poggioli G, Schreiber S, Talbot IC, Nicholls RJ, Gionchetti P, Campieri M, Kamm MA (2004). Once daily high dose probiotic therapy (VSL#3) for maintaining remission in recurrent or refractory pouchitis. Gut.

[B12] Kruis W, Schutz E, Fric P, Fixa B, Judmaier G, Stolte M (1997). Double-blind comparison of an oral Escherichia coli preparation and mesalazine in maintaining remission of ulcerative colitis. Aliment Pharmacol Ther.

[B13] Rembacken BJ, Snelling AM, Hawkey PM, Chalmers DM, Axon AT (1999). Non-pathogenic Escherichia coli versus mesalazine for the treatment of ulcerative colitis: a randomised trial. Lancet.

[B14] Kruis W, Fric P, Pokrotnieks J, Lukas M, Fixa B, Kascak M, Kamm MA, Weismueller J, Beglinger C, Stolte M, Wolff C, Schulze J (2004). Maintaining remission of ulcerative colitis with the probiotic Escherichia coli Nissle 1917 is as effective as with standard mesalazine. Gut.

[B15] Malchow HA (1997). Crohn's disease and Escherichia coli. A new approach in therapy to maintain remission of colonic Crohn's disease?. J Clin Gastroenterol.

[B16] Gupta P, Andrew H, Kirschner BS, Guandalini S (2000). Is lactobacillus GG helpful in children with Crohn's disease? Results of a preliminary, open-label study. J Pediatr Gastroenterol Nutr.

[B17] Guandalini S (2002). Use of Lactobacillus-GG in paediatric Crohn's disease. Dig Liver Dis.

[B18] Prantera C, Scribano ML (2002). Probiotics and Crohn's disease. Dig Liver Dis.

[B19] Guslandi M, Mezzi G, Sorghi M, Testoni PA (2000). Saccharomyces boulardii in maintenance treatment of Crohn's disease. Dig Dis Sci.

[B20] Plein K, Hotz J (1993). Therapeutic effects of Saccharomyces boulardii on mild residual symptoms in a stable phase of Crohn's disease with special respect to chronic diarrhea--a pilot study. Z Gastroenterol.

[B21] Ballongue J, Schumann C, Quignon P (1997). Effects of lactulose and lactitol on colonic microflora and enzymatic activity. Scand J Gastroenterol Suppl.

[B22] Kanauchi O, Iwanaga T, Mitsuyama K (2001). Germinated barley foodstuff feeding. A novel neutraceutical therapeutic strategy for ulcerative colitis. Digestion.

[B23] Bouhnik Y, Raskine L, Simoneau G, Vicaut E, Neut C, Flourie B, Brouns F, Bornet FR (2004). The capacity of nondigestible carbohydrates to stimulate fecal bifidobacteria in healthy humans: a double-blind, randomized, placebo-controlled, parallel-group, dose-response relation study. Am J Clin Nutr.

[B24] Madsen KL, Doyle JS, Jewell LD, Tavernini MM, Fedorak RN (1999). Lactobacillus species prevents colitis in interleukin 10 gene-deficient mice. Gastroenterology.

[B25] Rumi G, Tsubouchi R, Okayama M, Kato S, Mozsik G, Takeuchi K (2004). Protective effect of lactulose on dextran sulfate sodium-induced colonic inflammation in rats. Dig Dis Sci.

[B26] Riemann JF (2001). [Guidelines of the DGVS. Clinical diagnosis. German Society of Digestive and Metabolic Diseases]. Z Gastroenterol.

[B27] von Herbay A (2001). [Guidelines of the DGVS. Histopathological diagnosis. German Society of Digestive and Metabolic Diseases]. Z Gastroenterol.

[B28] Petritsch W, Feichtenschlager T, Gasche C, Hinterleitner T, Judmaier G, Knoflach P, Moser G, Offner F, Peer G, Simbrunner I (1998). [Diagnosis in chronic inflammatory bowel diseases--report of the Austrian Chronic Inflammatory Bowel Disease Study Group]. Acta Med Austriaca.

[B29] Scholmerich J (2001). [Guidelines of the DGVS. Chronic active course. German Society of Digestive and Metabolic Diseases]. Z Gastroenterol.

[B30] Fleig WE (2001). [Guidelines of the DGVS. Acute process. German Society of Digestive and Metabolic Diseases]. Z Gastroenterol.

[B31] Reissmann A, Fleig W (2002). [Therapy of Crohn disease according to the guidelines of the German Society for the treatment of digestive and metabolic diseases]. Z Arztl Fortbild Qualitatssich.

[B32] Rachmilewitz D (1989). Coated mesalazine (5-aminosalicylic acid) versus sulphasalazine in the treatment of active ulcerative colitis: a randomised trial. BMJ.

[B33] Shine B, Berghouse L, Jones JE, Landon J (1985). C-reactive protein as an aid in the differentiation of functional and inflammatory bowel disorders. Clin Chim Acta.

[B34] Best WR, Becktel JM, Singleton JW, Kern F (1976). Development of a Crohn's disease activity index. National Cooperative Crohn's Disease Study. Gastroenterology.

[B35] Goebell H (1988). European cooperative Crohn's disease study (ECCDS): Evaluation of different activity indices and development of a new severity-activity index (SAI). Inflamm Bowel Dis.

[B36] Sandborn WJ, Feagan BG, Hanauer SB, Lochs H, Lofberg R, Modigliani R, Present DH, Rutgeerts P, Scholmerich J, Stange EF, Sutherland LR (2002). A review of activity indices and efficacy endpoints for clinical trials of medical therapy in adults with Crohn's disease. Gastroenterology.

[B37] Hauser W, Dietz N, Grandt D, Steder-Neukamm U, Janke KH, Stein U, Stallmach A (2004). Validation of the inflammatory bowel disease questionnaire IBDQ-D, German version, for patients with ileal pouch anal anastomosis for ulcerative colitis. Z Gastroenterol.

[B38] BARON JH, CONNELL AM, LENNARD-JONES JE (1964). VARIATION BETWEEN OBSERVERS IN DESCRIBING MUCOSAL APPEARANCES IN PROCTOCOLITIS. Br Med J.

[B39] Rutgeerts P, Geboes K, Vantrappen G, Beyls J, Kerremans R, Hiele M (1990). Predictability of the postoperative course of Crohn's disease. Gastroenterology.

[B40] Cooke WT, Prior P (1984). Determining disease activity in inflammatory bowel disease. J Clin Gastroenterol.

[B41] Seibold F (2003). [Laboratory diagnosis in inflammatory bowel disease]. Ther Umsch.

[B42] Geboes K, Riddell R, Ost A, Jensfelt B, Persson T, Lofberg R (2000). A reproducible grading scale for histological assessment of inflammation in ulcerative colitis. Gut.

[B43] Mason DY, Cordell J, Brown M, Pallesen G, Ralfkiaer E, Rothbard J, Crumpton M, Gatter KC (1989). Detection of T cells in paraffin wax embedded tissue using antibodies against a peptide sequence from the CD3 antigen. J Clin Pathol.

[B44] Riordan SM, McIver CJ, Wakefield D, Duncombe VM, Thomas MC, Bolin TD (2001). Small intestinal mucosal immunity and morphometry in luminal overgrowth of indigenous gut flora. Am J Gastroenterol.

[B45] Sasaki Y, Tanaka M, Kudo H (2002). Differentiation between ulcerative colitis and Crohn's disease by a quantitative immunohistochemical evaluation of T lymphocytes, neutrophils, histiocytes and mast cells. Pathol Int.

[B46] Mayer L, Eisenhardt D, Salomon P, Bauer W, Plous R, Piccinini L (1991). Expression of class II molecules on intestinal epithelial cells in humans. Differences between normal and inflammatory bowel disease. Gastroenterology.

[B47] Beaugerie L, Berenbaum F, Berrebi D, Gendre JP, Prier A, Kaplan G, Chatelet FP (2001). Chronic use of non-steroidal anti-inflammatory drugs does not alter colonic mucosa of patients without diarrhoea. Aliment Pharmacol Ther.

[B48] Fellermann K, Wehkamp J, Herrlinger KR, Stange EF (2003). Crohn's disease: a defensin deficiency syndrome?. Eur J Gastroenterol Hepatol.

[B49] Szilagyi A, Rivard J, Shrier I (2002). Diminished efficacy of colonic adaptation to lactulose occurs in patients with inflammatory bowel disease in remission. Dig Dis Sci.

[B50] Cellier C, Sahmoud T, Froguel E, Adenis A, Belaiche J, Bretagne JF, Florent C, Bouvry M, Mary JY, Modigliani R (1994). Correlations between clinical activity, endoscopic severity, and biological parameters in colonic or ileocolonic Crohn's disease. A prospective multicentre study of 121 cases. The Groupe d'Etudes Therapeutiques des Affections Inflammatoires Digestives. Gut.

[B51] Carbonnel F, Lavergne A, Lemann M, Bitoun A, Valleur P, Hautefeuille P, Galian A, Modigliani R, Rambaud JC (1994). Colonoscopy of acute colitis. A safe and reliable tool for assessment of severity. Dig Dis Sci.

[B52] Riley SA, Mani V, Goodman MJ, Dutt S, Herd ME (1991). Microscopic activity in ulcerative colitis: what does it mean?. Gut.

[B53] Bouhnik Y, Neut C, Raskine L, Michel C, Riottot M, Andrieux C, Guillemot F, Dyard F, Flourie B (2004). Prospective, randomized, parallel-group trial to evaluate the effects of lactulose and polyethylene glycol-4000 on colonic flora in chronic idiopathic constipation. Aliment Pharmacol Ther.

